# A new redescription of *Richtersius coronifer*, supported by transcriptome, provides resources for describing concealed species diversity within the monotypic genus *Richtersius* (Eutardigrada)

**DOI:** 10.1186/s40851-020-0154-y

**Published:** 2020-02-05

**Authors:** Daniel Stec, Łukasz Krzywański, Kazuharu Arakawa, Łukasz Michalczyk

**Affiliations:** 1 Department of Entomology, Institute of Zoology and Biomedical Research, Jagiellonian University, Gronostajowa 9, 30-387 Kraków, Poland; 2 Institute for Advanced Biosciences, Keio University, Mizukami 246-2, Kakuganji, Tsuruoka, Yamagata, Japan

**Keywords:** Biodiversity, Transcriptome, Integrative taxonomy, Limno-terrestrial life cycle, Redescription, Spitsbergen

## Abstract

*Richtersius coronifer*, the nominal species for the family Richtersiidae and a popular laboratory model, exemplifies a common problem in modern tardigrade taxonomy. Despite undeniable progress in the field, many old and incomplete descriptions of taxa hinder both species delimitation and the estimation of species diversity and distribution. Although for over a century this species has been recorded throughout the world, recent research indicates that records to date are likely to represent a species complex rather than a single cosmopolitan species. However, in order to recognise and name species diversity within the complex, an integrative redescription of the nominal species is first needed. Here, we describe an *R. coronifer* population collected from Spitsbergen, i.e., one of the two localities mentioned in the original description, with detailed morphological and morphometric data associated with standard DNA sequences of four standard genetic markers (18S rRNA, 28S rRNA, ITS-2, and COI) and supported by transcriptome sequencing. We propose replacement of the neotype designated in 1981 by Maucci and Ramazzotti, as it is impossible to verify whether the existing neotype is conspecific with specimens studied by Richters in 1903 and 1904. Finally, using newly obtained cytochrome c oxidase subunit I (COI) sequences of populations from Spitsbergen, Italy, Poland, and Greece together with sequences deposited in GenBank (China, Greenland, Italy, Mongolia), we performed genetic species delimitation, which indicated seven distinct potential species within the genus *Richtersius*, in addition to the nominal taxon. This study marks a starting point for further research on the taxonomy of and species diversity within the genus. Moreover, this work has the potential to be the first tardigrade redescription to provide both genetic barcodes and a transcriptome of the species in question.

## Background

The phylum Tardigrada consists of small invertebrates which inhabit terrestrial and aquatic habitats [1], with about 1300 species discovered so far [2–4]. The tardigrade taxonomy is considered as challenging due to the small number of taxonomically informative characters and microscopic size of these animals [5]. In the past, the limited analytical tools (e.g., low-quality light microscopes, lack of electron microscopy and DNA sequencing) combined with the notion that tardigrade species exhibit wide morphological variability and are cosmopolitan (e.g. see [6]) led to considerable underestimations of tardigrade species diversity. This was due to the fact that, in most cases, only individuals characterised by obvious morphological traits were identified as representing new taxa, whereas species exhibiting minor differences went undetected [7]. Thus, many early-described species were considered cosmopolitan, whereas now they are recognised as complexes of species comprising morphologically very similar taxa, possibly each with a limited geographic range [7–11]. The understanding of tardigrade diversity started to change when taxonomists recognised that intraspecific morphological variability is, in many cases, much more limited than previously assumed (e.g. [5, 12]). This realization has resulted in an increased number of descriptions of species based on subtler traits. However, the greatest promise in unravelling tardigrade species diversity comes with the growing use of the tools of integrative taxonomy, as genetic analyses enable the detection of cryptic and pseudocryptic taxa that fly beneath the radar of classical taxonomy. Thus, thanks to constantly decreasing costs of DNA barcoding, we are now in an important moment in tardigrade taxonomy, and may be on the verge of discovering tardigrade species diversity in its fullness. Nevertheless, despite the increasing resolution in the detection of tardigrade species diversity, out-dated descriptions and the lack of type material for the nominal taxa remain significant obstacles to species descriptions within a given group (e.g. a genus or a species complex).

One such nominal species is *Richtersius coronifer* (Richters, 1903) [13], which was originally described as *Macrobiotus coronifer* based on populations collected in Klaas Billen Bay (now renamed as Billefjorden) in Svalbard (a Norwegian Arctic archipelago) and Tromsø (a city in continental Norway), as a yellow tardigrade with two macroplacoids, large claws with accessory points and lunules with distinct teeth (the description and the information of the species was provided once again by Richters [14]). Such a general description, which provides only the most evident morphological characteristics, does not allow species identification under the standards of modern tardigrade taxonomy. Moreover, the identification of *R. coronifer* is further impeded by the fact that the original Richters’ type series is no longer available. Maucci & Ramazzotti [15] attempted to overcome this problem by redescribing *Macrobiotus coronifer*, establishing a neotype and transferring this species to a newly erected genus, *Adorybiotus*. Later, Pilato & Binda [16] redefined the genus *Adorybiotus* Maucci & Ramazzotti, 1981 [15] and established a new monotypic genus *Richtersia* Pilato & Binda, 1987 [16]; they again changed the name, to *Richtersius* Pilato & Binda, 1989 [17], two years later. From that time, *Richtersius coronifer* (Richters, 1903) [13] remains the only member of the genus *Richtersius*, and it is also a nominal species for a recently erected family Richtersiidae Guidetti, Rebecchi, Bertolani, Jönsson, Kristensen & Cesari, 2016 [18].

Although the neotype of *R. coronifer* is available, there are several important reasons to question the validity of that designation, highlighting the need to replace the neotype with new material. First, Maucci & Ramazzotti [15] based their redescription on specimens collected from a different locality than those studied by Richters [13]. Specifically, they used a population from Bodø in continental Norway, which is over 1200 km distant from Klaas Billen Bay and 325 km from Tromsø. Second, the microscope slides with specimens from the Bodø population most likely comprise two *Richtersius* species (Roberto Guidetti, pers. com.); thus, the 1981 redescription is not based on a single species, and it is not possible to ascertain which of the two species was used to establish the neotype. Third, previous studies [18–20] and our analyses presented here (see Results) show that there are at least four *Richtersius* species present in continental Europe. Whether the neotype is indeed conspecific with specimens from the Tromsø locality found by Richters [13] is thus subject to reasonable doubt. Maucci & Ramazzotti [15] were almost certainly not aware of the high species diversity in the genus, which became apparent only recently thanks to the use of genetic data. At the time of the redescription, *Richtersius* was considered monospecific and *R. coronifer* was assumed to be cosmopolitan. In fact, the detection of four *Richtersius* species in continental Europe, none of which are found in the Arctic, combined with the poor understanding of tardigrade species diversity at the beginning of the twentieth century strongly suggests that the original description of *R. coronifer* by Richters [13] was most likely based on more than one species. If that is indeed the case, specimens from either Klaas Billen Bay or Tromsø could be used to establish the neotype. In addition to the problems with the designation of the current neotype, it should be noted that there are no DNA sequences associated with the existing neotype; thus, the usefulness of the existing questionable neotype in species differentiation in the genus is extremely limited.

Given that *R. coronifer* is the type species for the genus, the lack of a modern description impedes the exploration of species diversity within *Richtersius*. Moreover, recently, a *Richtersius* species, identified as “*R. coronifer*”, has become a popular model organism in physiological studies on cryptobiosis (e.g .[21–41]). This further stresses the importance of redescribing *R. coronifer*, as this will allow a detailed and correct identification of populations used as laboratory models. Thus, to remove the obstacle and open doors to descriptions of other *Richtersius* species, we performed an integrative redescription of *R. coronifer* using a population collected in the Klaas Billen Bay, one of the localities studied by Richters [13] in the original description of the species. The redescription comprises detailed morphological and morphometric data, obtained using phase and Nomarski contrast (PCM, NCM) light microscopy, as well as scanning electron microscopy (SEM). The phenotypic characterisation is associated with molecular data in form of DNA sequences of four standard genetic markers, three nuclear (18S rRNA, 28S rRNA, ITS-2) one mitochondrial (COI), and—for the first time in the history of tardigrade species redescriptions—further supported with a transcriptome. Alongside the redescription, we constructed the COI phylogeny of the five newly discovered European populations that all fit the general original description of *R. coronifer*, together with previously published COI sequences for the genus *Richtersius*. Finally, we performed genetic species delimitation analysis, which indicates the presence of at least seven new putative species within the genus.

## Materials and methods

### Samples and specimens

As stated in the Introduction, *Richtersius coronifer* was originally described by Richters [13] as *Macrobiotus coronifer* from Klaas Billen Bay (= Billefjorden) in Svalbard and Tromsø, and later redescribed by Maucci & Ramazzotti [15], who designated a neotype from a different population from continental Norway. However, it is impossible to establish whether the three reported populations represent the same, or distinct but morphologically similar, species. Moreover, the microscope slide with specimens from the Bodø population comprises two morphologically distinct species (Roberto Guidetti, pers. com.). Taking into consideration the discussion above, as well as our present findings that multiple morphologically very similar species exist in the genus *Richtersius*, a request will be prepared and submitted to the International Commission of Zoological Nomenclature asking to set aside, under its plenary power [Art. 81], the existing neotype designated by Maucci & Ramazzotti [15] and to designate a new neotype from a population collected in one of the localities mentioned in the original description of the species and examined in the present study (i.e. Billefjorden, Svalbard, Norway, see Table 1 for more details) in order to promote stability of the nomenclature for the genus *Richtersius*.

**Table 1:** Information on moss samples with the *Richtersius* populations analysed in our study

Sample/population code	Species	Locality	Coordinates and altitude	Collector	Analyses performed and numbers specimens used			
					LCM	SEM	DNA	SEX
**NO.385**	***Richtersius coronifer s.s.***	**Norway, Svalbard, Billefjorden, Brucebyen**	**78°38′13″N** **16°46′07″E** **15 m asl**	**Michala Bryndová**	**260a** **45 h** **236e**	**15a** **5 h** **15e**	**4a** **0 h** **0e**	**60a** **0 h** **0e**
IT.120	*Richtersius* sp. 4	Italy, Lago di Teleccio, Val di Piantonetto	45°28′55″N 7°22′22″E 1830 m asl	Witold Morek, Piotr Gąsiorek	50a 10 h 41e	0a 0 h 0e	4a 0 h 0e	0a 0 h 0e
PL.246	*Richtersius* sp. 4	Poland, Tatrzański National Park, Kościeliska Valley	49°14′22″N 19°51′46″E 1083 m asl	Piotr Gąsiorek	312a 58 h 50e	0a 0 h 0e	4a 0 h 0e	0a 0 h 0e
IT.317	*Richtersius* sp. 6	Italy, Sardegna, Genna Silana	40°09′04″N 9°30′23″E 1047 m asl	Peter Degma	20a 0 h 21e	0a 0 h 0e	3a 0 h 0e	0a 0 h 0e
GR.008	*Richtersius* sp. 7	Greece, Crete, Omalos, Chania	35°16′16″N 23°57′41″E 456 m asl	Małgorzata Mitan, Małgorzata Osielczak	116a 11 h 59e	0a 0 h 0e	4a 0 h 0e	0a 0 h 0e

Bold font indicates the *Richtersius coronifer s.s.* which is proposed as the new neotype population. Analyses performed: LCM – light contrast microscopy observations, SEM – scanning electron microscope observations, DNA – DNA sequencing, SEX – sex determination by aceto-orcein staining; a = number of adults, h = number of hatchlings (first instars), e = number of eggs

Additionally, in order to test whether the genus *Richtersius* is monotypic or comprises multiple species, we analysed genetic data of four newly found populations isolated from samples collected from four European localities, which could be identified as *R. coronifer* according to the original description (see Table 1 for details). All samples were processed following a protocol described in detail in Stec et al. [42]. Depending on the numbers of available animals and eggs, the specimens were divided in up to four groups, which were used for different analyses (see Table 1 for details): (i) imaging by light microscopy (external and internal morphology and morphometry), (ii) imaging by scanning electron microscopy (SEM fine external morphology and buccal apparatus anatomy, the Billefjorden population only), (iii) DNA extraction and sequencing, and (iv) aceto-orcein staining (to test for the presence of males, the Billefjorden population only), but adults freshly mounted in Hoyer’s medium from all populations were examined for spermatozoa (sperm in eutardigrades are typically detectable up to 24 h after mounting in Hoyer’s medium [43];).

### Microscopy and imaging

Specimens for light microscopy were mounted on microscope slides in a small drop of Hoyer’s medium and secured with a cover slip, following the protocol by Morek et al. [44]. Slides were examined under an *Olympus BX53* light microscope with phase and Nomarski interference contrasts (PCM and NCM, respectively; together termed here as light contrast microscopy, LCM), associated with an *Olympus DP74* digital camera. In order to obtain clean and extended specimens for SEM, tardigrades and eggs were processed according to the protocol by Stec et al. [42]. Bucco-pharyngeal apparatuses were extracted following the protocol of Eibye-Jacobsen [45] as modified by Gąsiorek et al. [46]. All specimens were examined under high vacuum in a Versa 3D DualBeam scanning electron microscope (SEM) at the ATOMIN facility of the Jagiellonian University, Kraków, Poland. The Billefjorden population was also examined for the presence of males with aceto-orcein staining [47], following Stec et al. [48]. All figures were assembled in *Corel Photo-Paint X6*, ver. 16.4.1.1281. For structures that could not be captured in a single photograph, a stack of 2–6 images were taken with an equidistance of ca. 0.2 μm and assembled manually into a single deep-focus image.

### Morphometrics and morphological nomenclature

All measurements are given in micrometres (μm). Sample size was adjusted following recommendations by Stec et al. [49]. Structures were measured only if their orientation was suitable. Body length was measured from the anterior extremity to the end of the body, excluding the hind legs. The terminology used to describe oral cavity armature and egg shell morphology follows Michalczyk & Kaczmarek [50], Kaczmarek & Michalczyk [51] and Guidetti et al. [18]. Macroplacoid length sequence is given following Kaczmarek et al. [52]. Buccal tube length and the level of the stylet support insertion point were measured according to [12]. The *pt* index is the ratio of the length of a given structure to the length of the buccal tube expressed as a percentage [12]. Buccal tube width was measured as the external and internal diameter at the level of the stylet support insertion point. Heights of claw branches were measured following Kaczmarek & Michalczyk [51], i.e., from the base of the claw (i.e. excluding the lunulae) to the top of the branch, including accessory points. The claw common tract index (*cct*) is the proportion of the height of the common tract of the claw (measured from the claw base to the separation point between the first and the second branch) to the total claw height expressed as a percentage [18]. Distance between egg processes was measured as the shortest line connecting base edges of the two closest processes [51]. Morphometric data were handled using the “Parachela” ver. 1.7 template available from the Tardigrada Register [53]. Tardigrade taxonomy follows [18, 54, 55].

In search for new phenotypic criteria for species differentiation in the genus *Richtersius*, we measured six additional traits: cuticular pore density (PD, the number of pores per 2500 μm^2^ counted within a rectangle on the dorsal cuticle between legs III and IV), pore size (PS, measured as largest diameter) the number of teeth on external and internal lunules III (ExtT and IntT, respectively) and the number of teeth on anterior and posterior lunules IV (AntT and PosT, respectively). Additionally, we tested two morphometric traits that were shown by Guidetti et al. [18] to differentiate *Richtersius* populations studied therein. Specifically, we used the *pt* of the stylet support insertion point (SSIP) and claw common track index for external claws III (CCT). The measurements of each trait were conducted on 10 animals for each of the three of five genetically delimited species found in this study, represented by the following populations: NO.385 (the Billefjorden population), GR.008, and IT.120 (Table 1). For pore size, 10 pores each from 10 specimens per population were measured. The two remaining new populations were not used in this part of our study, since population PL.246 represented the same new species as the population IT.120 whereas the Sardinian population IT.317 comprised only several adults, meaning that the cuticular pores (present only in the first instar, termed here as “hatchlings”) could not be examined. A series of one-way ANOVA tests followed by post-hoc Tukey comparisons were used to examine the differences between mentioned populations in each trait independently (PD, ExtT, IntT, AntT, PosT). For pore size, a nested ANOVA with PS as dependent variable and two fixed factors, population and specimen nested in population, was run and further differences between populations were tested with Tukey post-hoc test. The statistics were computed using STATISTICA ver.13.0 (Tibco, Poland). All raw measurements and computed statistics are given in supplementary materials (Additional file 1).

### Genotyping

Genomic DNA for barcoding was extracted from individual animals following a *Chelex® 100* resin (*BioRad*) extraction method by Casquet et al. [56] with modifications described in detail in Stec et al. [42]. We sequenced four DNA fragments, three nuclear (18S rRNA, 28S rRNA, ITS2) and one mitochondrial (COI) from 3 to 4 individuals per each of the five analysed populations. All fragments were amplified and sequenced according to the protocols described in Stec et al. [42]; primers and original references for specific PCR programs are listed in Table 2. Sequencing products were read with the *ABI 3130xl* sequencer at the Molecular Ecology Lab, Institute of Environmental Sciences of the Jagiellonian University, Kraków, Poland. Sequences were processed in *BioEdit* ver. 7.2.5 [63]. Single DNA sequences per haplotype for each analysed population were submitted to GenBank.

**Table 2:** Primers and references for PCR protocols for amplification of the four DNA fragments sequenced in the study

DNA marker	Primer name	Primer direction	Primer sequence (5′-3′)	Primer source	PCR programme
18S rRNA	18S_Tar_Ff1	forward	AGGCGAAACCGCGAATGGCTC	[57]	[58]
	18S_Tar_Rr1	reverse	GCCGCAGGCTCCACTCCTGG		
28S rRNA	28S_Eutar_F	forward	ACCCGCTGAACTTAAGCATAT	[59, 60]	[60]
	28SR0990	reverse	CCTTGGTCCGTGTTTCAAGAC		
ITS-2	ITS2_Eutar_Ff	forward	CGTAACGTGAATTGCAGGAC	[7]	[7]
	ITS2_Eutar_Rr	reverse	TCCTCCGCTTATTGATATGC		
COI	COI_Para_F	forward	TTTCAACAAACCACAAAGATATYGG	[61]	[9]
	COI_Eutar_Rr	reverse	TAAACTTCTGGGTGACCRAARAAYCA		
	LCO1490	forward	GGTCAACAAATCATAAAGATATTGG	[62]	
	COI_Mac_Rr	reverse	TGTTGGTATARAATWGGGTC	[101]	

### Comparative genetic analysis

For molecular comparisons, all published sequences of the 18S rRNA and COI markers of suitable length, for the genus *Richtersius* were downloaded from GenBank ([18, 20, 54, 64–66] and Li & Xiao, unpublished). There were no ITS-2 sequences available in GenBank for the genus *Richtersius*, whereas the 28S rRNA sequences published by Guidetti et al. [18] represent a fragment that is different from our sequences. Thus, for ITS-2 as well as 28S rRNA we analysed only the data from the five populations examined in this study. Two options, nucleotide BLAST and blastx, of the Basic Local Alignment Search Tool [67], showed that two sequences (GU237485, GU339056) by Li & Xiao (unpublished) represent the genus *Richtersius*, with the second being mistakenly labelled as “*Paramacrobiotus richtersi*”. The sequences were aligned using the default settings (in the case of COI) and the Q-INS-I method (in the case of ribosomal markers: 18S rRNA, 28S rRNA and ITS-2) of *MAFFT* version 7 [68, 69] and manually checked against non-conservative alignments in *BioEdit*. Then, the aligned sequences were trimmed to: 765 (18S rRNA), 765 (28S rRNA), 440 (ITS-2), 561 (COI), bp. All COI sequences were translated into protein sequences in *MEGA7* version 7.0 [70] to check against pseudogenes. Uncorrected pairwise distances, as well as mean genetic distances within and between genetically delimited species, were calculated using *MEGA7*.

### Phylogenetic analysis

To construct phylogenetic trees, we used all COI sequences of *Richtersius* populations genotyped in this study (18 sequences; Tables 1 and 3), and *Richtersius* COI sequences available in GenBank (22 sequences; for details see section *Comparative molecular analysis* above) with *Macrobiotus papei* Stec, Kristensen & Michalczyk, 2018 [71] as the outgroup. The sequences were aligned using the default settings of MAFFT version 7 [68, 69], then edited and checked manually in *BioEdit*. The obtained alignment was trimmed to 561 bp. Using PartitionFinder version 2.1.1 [72] under the Bayesian Information Criterion (BIC), the best scheme of partitioning and substitution models were chosen for posterior phylogenetic analysis. We ran the analysis to test all possible models implemented in MrBayes and RAxML software (see below for specific references). As the COI is a protein coding gene, before partitioning, we divided our alignments of this marker into three data blocks representing separate three-codon positions. As a best-fit partitioning scheme, PartitionFinder suggested to always retain all predefined partitions separately. The specific substitution models suggested for our data set and partitions were GTR + I for all partitions for RAxML and for MrBayes SYM + I, F81 + G, HKY + G for 1st, 2nd and 3rd codon position respectively.

**Table 3:** Measurements [in μm] and *pt* values of selected morphological structures of animals of *Richtersius coronifer s.s.* (Richters, 1903) from the Billefjorden population. (Specimens mounted in Hoyer’s medium; N – number of specimens/structures measured, RANGE refers to the smallest and the largest structure among all measured specimens; SD – standard deviation; * – proposed new neotype)

Character	*N*	Range						Mean		SD		Neotype*	
		μm			*pt*			μm	*pt*	μm	*pt*	μm	*pt*
Body length	30	499	–	1027	*701*	*–*	*1125*	771	*898*	164	*117*	984	*1057*
Buccopharyngeal tube													
Buccal tube length	30	68.5	–	100.5		–		85.2	*–*	10.0	*–*	93.1	*–*
Stylet support insertion point	30	51.2	–	74.4	*72.1*	*–*	*75.6*	63.0	*74.0*	7.2	*1.1*	68.8	*73.9*
Buccal tube external width	30	5.6	–	10.5	*7.6*	*–*	*11.0*	8.0	*9.4*	1.3	*1.0*	9.5	*10.2*
Buccal tube internal width	30	1.2	–	3.5	*1.7*	*–*	*3.8*	1.9	*2.3*	0.4	*0.5*	1.9	*2.0*
Ventral lamina length	30	32.0	–	47.6	*40.1*	*–*	*51.3*	39.3	*46.2*	4.8	*3.0*	43.3	*46.5*
Placoid lengths													
Macroplacoid 1	30	7.5	–	15.5	*10.2*	*–*	*15.9*	11.9	*13.9*	2.5	*1.8*	11.4	*12.2*
Macroplacoid 2	30	6.2	–	11.5	*8.7*	*–*	*12.6*	8.9	*10.4*	1.5	*1.1*	10.1	*10.8*
Macroplacoid row	30	14.8	–	28.5	*20.8*	*–*	*31.3*	22.2	*25.9*	3.7	*2.5*	22.9	*24.6*
Claw 1 heights													
External base	25	9.2	–	17.6	*13.0*	*–*	*20.6*	14.0	*16.5*	2.5	*1.8*	16.8	*18.0*
External primary branch	25	20.9	–	31.4	*27.4*	*–*	*36.7*	26.3	*31.2*	3.0	*2.4*	28.7	*30.9*
External secondary branch	24	12.1	–	25.4	*16.9*	*–*	*30.9*	17.7	*20.8*	3.6	*3.0*	22.1	*23.7*
External base/primary branch (*cct*)	25	42.0	–	59.2		–		53.0	*–*	5.1	*–*	58.5	*–*
Internal base	25	8.6	–	16.6	*12.2*	*–*	*19.3*	13.4	*16.0*	2.3	*1.7*	12.8	*13.7*
Internal primary branch	26	20.8	–	30.8	*25.2*	*–*	*36.5*	26.6	*31.5*	2.5	*2.6*	26.1	*28.0*
Internal secondary branch	25	10.7	–	21.5	*14.7*	*–*	*25.9*	16.0	*19.0*	3.2	*2.7*	?	*?*
Internal base/primary branch (*cct*)	24	40.2	–	57.8		–		50.6	*–*	4.3	*–*	49.0	*–*
Claw 2 heights													
External base	25	9.6	–	19.0	*13.6*	*–*	*20.9*	14.5	*16.9*	2.7	*1.8*	15.4	*16.5*
External primary branch	29	23.2	–	33.7	*27.9*	*–*	*38.7*	28.5	*33.4*	3.0	*2.3*	30.5	*32.8*
External secondary branch	27	11.3	–	22.9	*15.5*	*–*	*25.0*	18.2	*21.0*	3.1	*2.2*	22.9	*24.6*
External base/primary branch (*cct*)	25	40.7	–	58.2		–		51.0	*–*	5.5	*–*	50.5	*–*
Internal base	26	9.0	–	18.2	*12.7*	*–*	*21.3*	14.6	*16.9*	2.5	*1.7*	15.6	*16.8*
Internal primary branch	28	23.7	–	35.2	*27.6*	*–*	*37.2*	28.9	*33.9*	3.1	*2.4*	30.1	*32.3*
Internal secondary branch	28	12.8	–	21.8	*16.5*	*–*	*25.5*	17.2	*20.0*	2.7	*1.8*	19.7	*21.2*
Internal base/primary branch (*cct*)	26	38.0	–	57.6		–		49.9	*–*	5.4	*–*	51.8	*–*
Claw 3 heights													
External base	22	9.7	–	18.4	*13.9*	*–*	*20.6*	14.7	*17.2*	2.7	*1.6*	17.5	*18.8*
External primary branch	25	24.0	–	40.7	*30.3*	*–*	*41.2*	29.1	*34.5*	4.4	*2.8*	31.4	*33.7*
External secondary branch	23	12.0	–	23.1	*17.5*	*–*	*25.3*	18.4	*21.6*	3.6	*2.4*	22.1	*23.7*
External base/primary branch (*cct*)	22	39.3	–	57.6		–		50.5	*–*	5.2	*–*	55.7	*–*
Internal base	22	9.8	–	20.6	*14.1*	*–*	*21.3*	14.1	*16.7*	2.8	*1.9*	15.8	*17.0*
Internal primary branch	22	22.6	–	42.4	*30.6*	*–*	*42.9*	28.8	*34.4*	4.4	*2.9*	31.2	*33.5*
Internal secondary branch	22	11.5	–	29.2	*15.7*	*–*	*31.7*	16.9	*20.0*	4.1	*3.3*	19.6	*21.1*
Internal base/primary branch (*cct*)	22	40.2	–	57.3		–		48.7	*–*	4.5	*–*	50.6	*–*
Claw 4 heights													
Anterior base	25	12.6	–	22.0	*15.1*	*–*	*25.7*	17.3	*20.5*	2.6	*2.4*	18.9	*20.3*
Anterior primary branch	26	29.1	–	44.4	*32.4*	*–*	*52.6*	35.8	*42.7*	4.0	*4.9*	39.8	*42.7*
Anterior secondary branch	25	13.6	–	28.0	*19.5*	*–*	*35.9*	20.5	*24.3*	3.8	*3.7*	23.3	*25.0*
Anterior base/primary branch (*cct*)	24	41.3	–	54.5		–		47.6	*–*	3.6	*–*	47.5	*–*
Posterior base	26	11.4	–	23.5	*16.1*	*–*	*24.3*	17.2	*20.5*	2.8	*2.1*	22.1	*23.7*
Posterior primary branch	28	27.2	–	42.2	*29.5*	*–*	*49.3*	35.0	*41.6*	3.8	*4.0*	40.0	*43.0*
Posterior secondary branch	27	12.8	–	27.3	*18.1*	*–*	*29.1*	20.0	*23.6*	4.0	*3.1*	25.9	*27.8*
Posterior base/primary branch (*cct*)	26	41.1	–	56.8		–		48.7	*–*	4.5	*–*	55.3	*–*

Bayesian inference (BI) marginal posterior probabilities were calculated using MrBayes v3.2 [73]. Random starting trees were used and the analysis was run for 8 million generations, sampling the Markov chain every thousand generations. An average standard deviation of split frequencies of < 0.01 was used as a guide to ensure the two independent analyses had converged. The program Tracer v1.6 [74] was then used to ensure Markov chains had reached stationarity and to determine the correct ‘burn-in’ for the analysis which was the first 10% of generations. The ESS values were greater than 200 and a consensus tree was obtained after summarising the resulting topologies and discarding the ‘burn-in’. Maximum-likelihood (ML) topologies were constructed using RAxML v8.0.19 [75]. Strength of support for internal nodes of ML construction was measured using 1000 rapid bootstrap replicates. All final consensus trees were viewed and visualised by FigTree v.1.4.3 available from http://tree.bio.ed.ac.uk/software/figtree.

### Transcriptome

For transcriptome sequencing and assembly, total RNA was extracted from a single specimen of tardigrade using Direct-zol RNA kit (Zymo Research) and was amplified using SMARTer Ultra Low Input RNA Kit for Sequencing v.3 (Clonetech). Illumina libraries were prepared using KAPA HyperPlus Kit (KAPA BIosystems), and the library was sequenced using a NextSeq 500 High Output Mode 75 cycles kit (Illumina) as single-end 75 bp layout. Protocols are detailed in Arakawa et al. [76] and Yoshida et al. [77]. Sequences were filtered for adapters and demultiplexed using the bcl2fastq v.2 software (Illumina), and were assembled de novo using Bridger software with default parameters [78]. Completeness of the assembly was assessed using BUSCO v.2/3 transcriptome mode with Eukaryote reference through gVolante server [79]. Gene content was compared with *Ramazzottius varieornatus* Bertolani & Kinchin, 1993 [80] and *Hypsibius exemplaris* Gąsiorek, Stec, Morek & Michalczyk, 2018 [61] genomes [81], as well as with *Drosophila melanogaster* Meigen, 1830 [82] and *Caenorhabditis elegans* (Maupas, 1900) [83] reference proteomes obtained from Flybase and Wormbase, respectively.

### Genetic species delimitation with COI sequences

To determine the number of putative species in our dataset, we analysed COI sequences with two independent genetic species delimitation methods, the Poisson Tree Processes (PTP) and Automatic Barcode Gap Discovery (ABGD).

The PTP method uses a non-ultrametric phylogenetic tree as the input data, based on which the switch from speciation to coalescent processes is modelled and then used to delineate primary species hypotheses [84]. For the PTP, we used BI and ML trees constructed as described above. In both cases, we discarded the outgroups to protect against eventual biases caused by distant relationship between the outgroup and the ingroup taxa. The calculations were conducted on the bPTP webserver (http://species.h-its.org/ptp), with 500,000 MCMC generations, thinning the set to 100, burning at 10% and performing a search for Maximum Likelihood and Bayesian solutions.

The ABGD method [85] uses an algorithmic calculation to detect the “barcode gap” within the distribution of calculated genetic pairwise distances. We used the ABGD web-server (www.abi.snv.jussieu.fr/public/abgd/abgdweb.html) and analysed the COI marker with default parameters.

### Data deposition

Raw morphometric measurements underlying the proposed redescription of *R. coronifer* are given in Supplementary Materials (Additional file 2) and are deposited in the Tardigrada Register [53] under www.tardigrada.net/register/0061.htm. The DNA sequences for the Billefjorden population and others examined populations are deposited in GenBank (https://www.ncbi.nlm.nih.gov/genbank, Table 3). Uncorrected pairwise distances are given in Supplementary Materials (Additional file 3). RNA-Seq data was deposited in NCBI SRA under BioProject PRJNA553097, and transcriptome assembly was deposited in FigShare under 10.6084/m9.figshare.8797184.

## Results

### Taxonomic account of the proposed neotype from the Billefjorden population

**Phylum:** Tardigrada Doyère, 1840 [86]

**Class:** Eutardigrada Richters, 1926 [87]

**Order:** Macrobiotoidea Thulin, 1928 [88, 89] in [55]

**Family:** Richtersiidae Guidetti, Rebecchi, Bertolani, Jönsson, Kristensen & Cesari, 2016 [18]

**Genus:** *Richtersius* Pilato & Binda, 1989 [17]

***Richtersius coronifer*** (Richters, 1903) [13]

(Tables 4 and 5, Figs. 1, 2, 3, 4, 5, 6, 7, and 8)

**Table 4:** Measurements [in μm] of eggs of *Richtersius coronifer s.s.* (Richters, 1903) from the Billefjorden population (eggs mounted in Hoyer’s medium; process base/height ratio is expressed as percentage; N – number of eggs/structures measured, RANGE refers to the smallest and the largest structure among all measured specimens; SD – standard deviation)

Character	N	Range			Mean	SD
Egg bare diameter	30	173.2	–	233.4	200.6	16.1
Egg full diameter	30	201.5	–	263.7	233.0	16.6
Process height	90	13.0	–	28.3	21.7	3.2
Process base width	90	2.7	–	6.9	4.6	0.9
Process base/height ratio	90	12%	–	33%	22%	5%
Inter-process distance	90	4.4	–	13.2	8.4	1.8
Number of processes on the egg circumference	30	60	–	77	67.1	4.5

**Table 5:** Sequences of the newly found *Richtersius* populations obtained in this study and used for molecular comparisons and phylogenetic analyses

Population	Species	18S rRNA	28S rRNA	ITS-2	COI
**NO.385**	***Richtersius coronifer s.s.***	**MH681760**	**MH681757**	**MH681763**	**MH676053**
IT.120	*Richtersius* sp. 4	MH681761	MH681758	MH681764	MH676054
PL.246	*Richtersius* sp. 4	MH681762	MH681759	MH681765	MH676055
IT.317	*Richtersius* sp. 6	MK211387	MK211385	MK211382–3	MK214326–8
GR.008	*Richtersius* sp. 7	MK211386	MK211384	MK211380–1	MK214323–5

Bold font indicates the *Richtersius coronifer s.s.* from the Billefjorden population. Please see Table 1 for geographic data

**Fig. 1:**
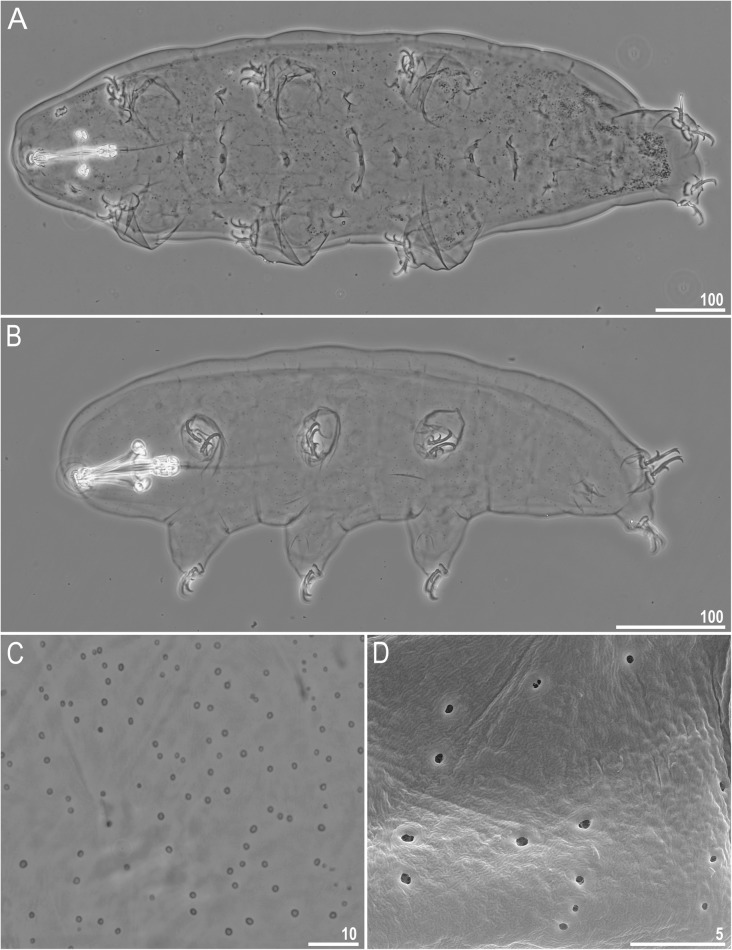
*Richtersius coronifer s.s.* (Richters, 1903) from the Billefjorden population. **a** adult habitus, dorso-ventral projection (this specimen will be proposed as a new neotype Stec et al. in prep., PCM). **b** Hatchling habitus, dorso-ventral projection (PCM). **c** Pores on the ventral cuticle of a hatchling (PCM). D. Pores on the dorsal cuticle of a hatchling (SEM). Scale bars in μm

**Fig. 2:**
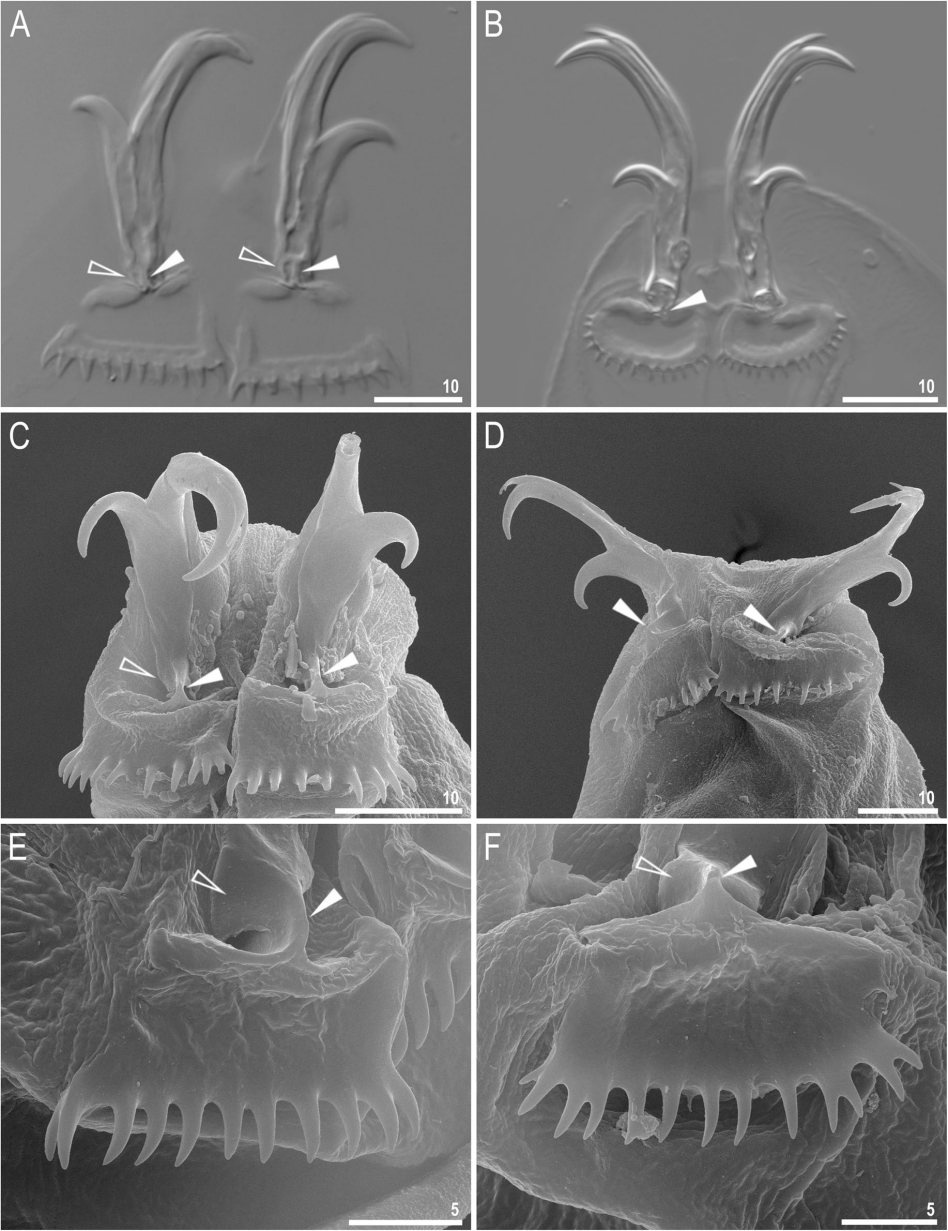
*Richtersius coronifer s.s.* (Richters, 1903) from the Billefjorden population, claws. **a**, **b** Claws III and IV seen under NCM. **c**, **d** Claws II and IV seen under SEM. **e**, **f** Lunules II and IV seen under SEM. Filled arrowheads indicate the laminar stalk connecting claw to the lunule, empty arrowheads indicate posterior lateral expansions. Scale bars in μm

**Fig. 3:**
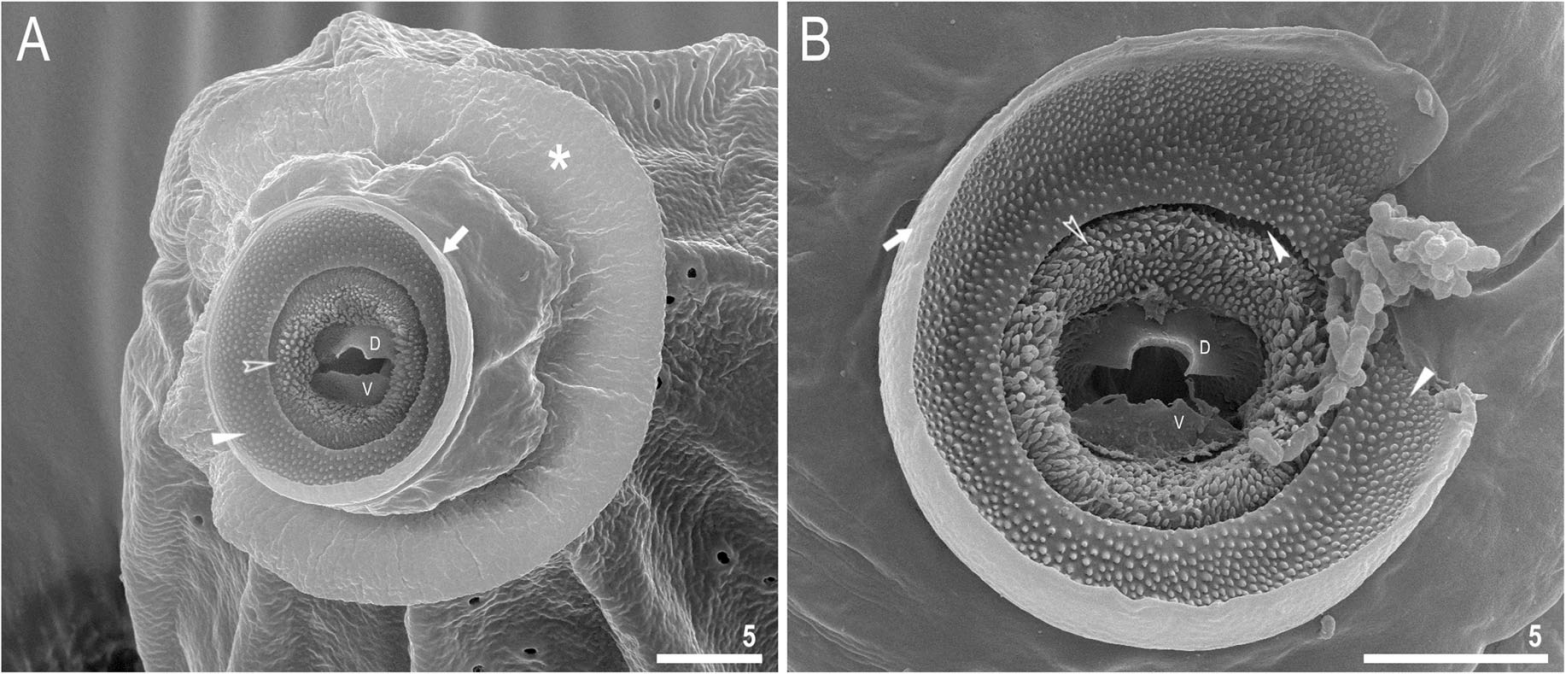
*Richtersius coronifer s.s.* (Richters, 1903) from the Billefjorden population, mouth seen in SEM. **a** head and mouth opening of a hatchling. **b** oral cavity armature of an adult. Asterisk indicates the circular sensory field, filled arrows indicate the peribuccal velum/lamina, filled arrowheads indicate the first band of teeth, filled indented arrowhead indicates the pre-mouth ventricle, empty indented arrowheads indicate the teeth of the second band, dorsal and ventral teeth of the third band are marked with D and V, respectively. Scale bars in μm

**Fig. 4:**
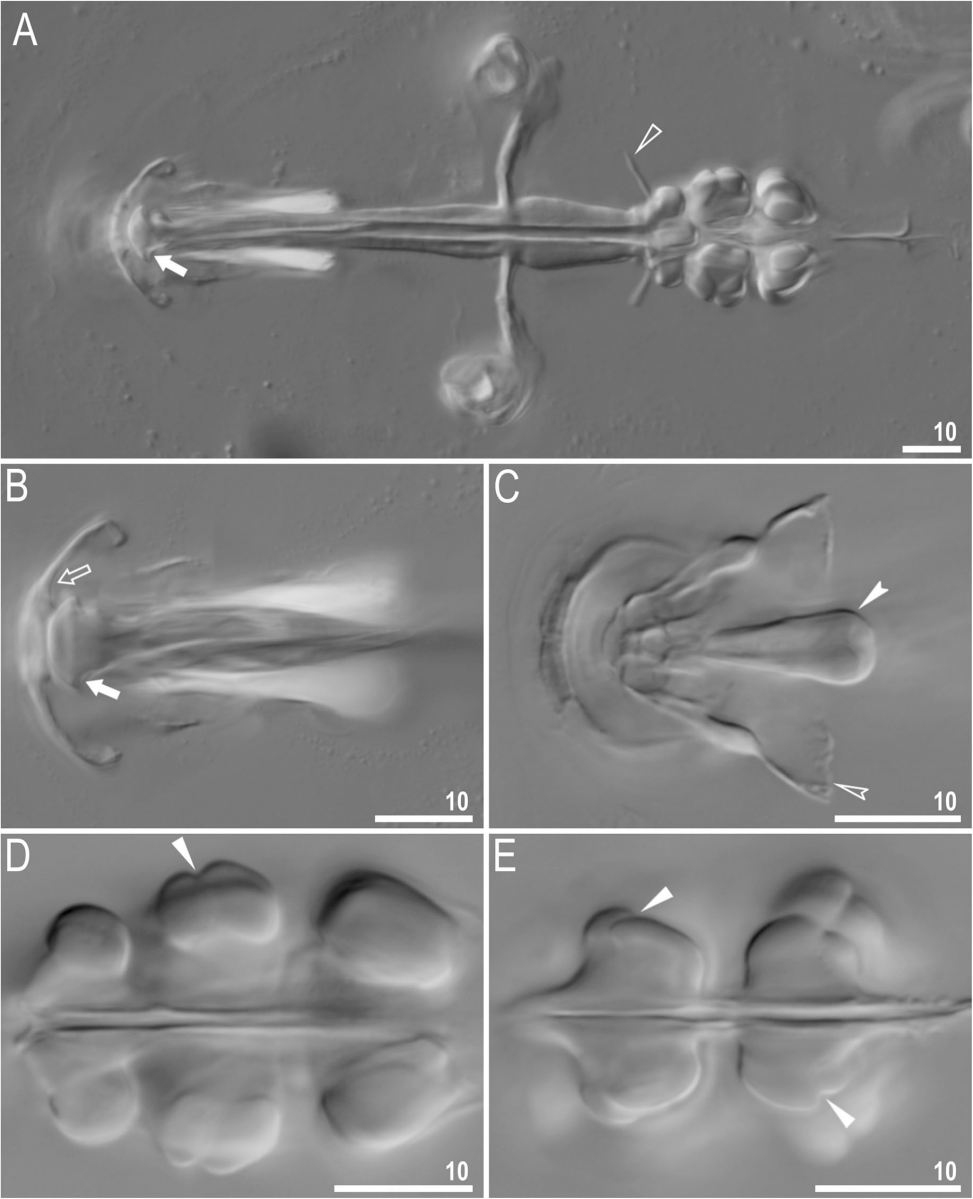
*Richtersius coronifer s.s.* (Richters, 1903) from the Billefjorden population, buccal apparatus seen in NCM. **a** Dorsal projection of the entire buccal apparatus. **b** Buccal crown, dorsal view **c** Buccal crown, ventral view. **d** Placoids, dorsal view. **e** Placoids ventral view. Filled arrows indicate the cuticular hook on the T-shaped apophysis, the empty arrow indicates the dorsal triangular apophysis, the filled indented arrowhead indicates the bulbous apophysis at the anterior end of the ventral lamina, the empty indented arrowhead indicates the ventral triangular apophysis, filled flat arrowheads indicate constrictions of macroplacoids, the empty flat arrowhead indicates dorsal spikes. Scale bars in μm

**Fig. 5:**
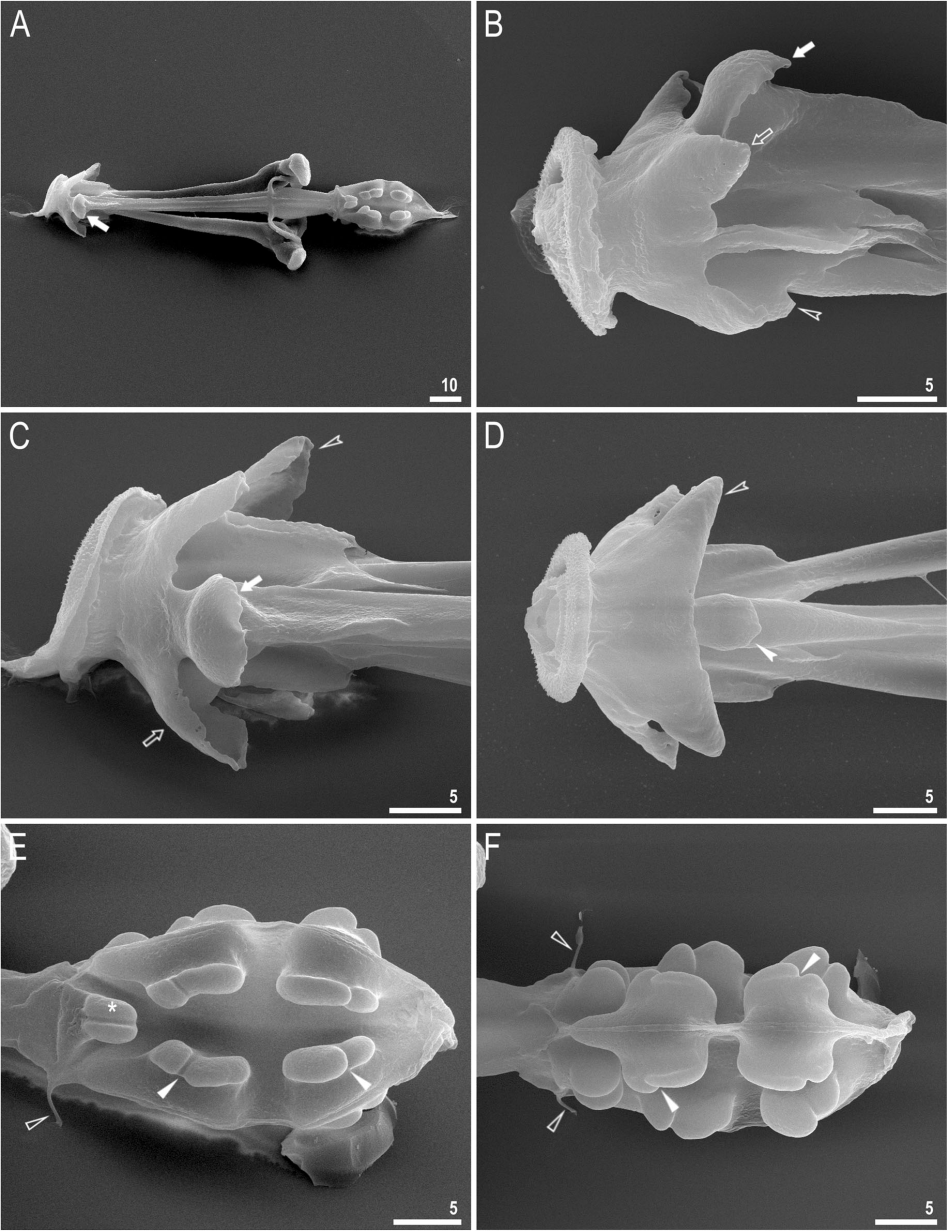
*Richtersius coronifer s.s.* (Richters, 1903) from the Billefjorden population, buccal apparatus seen under SEM. **a**. Dorsal view of the entire buccal apparatus. **b** Buccal crown, lateral view. **c** Buccal crown, dorsal view **d** Buccal crown, ventral view. **e** Placoids, dorsal view. **f** Placoids ventral view. Filled arrows indicate cuticular hook on T-shaped apophysis, empty arrow indicates dorsal triangular apophysis, filled indented arrowhead indicates bulbous apophysis on the begging of the ventral lamina, empty indented arrowhead indicates ventral triangular apophysis, filled arrowheads indicate constrictions of macroplacoids, empty arrowhead indicates dorsal spikes, asterisk indicates a bilobed apophysis in the pharynx. Scale bars in μm

**Fig. 6:**
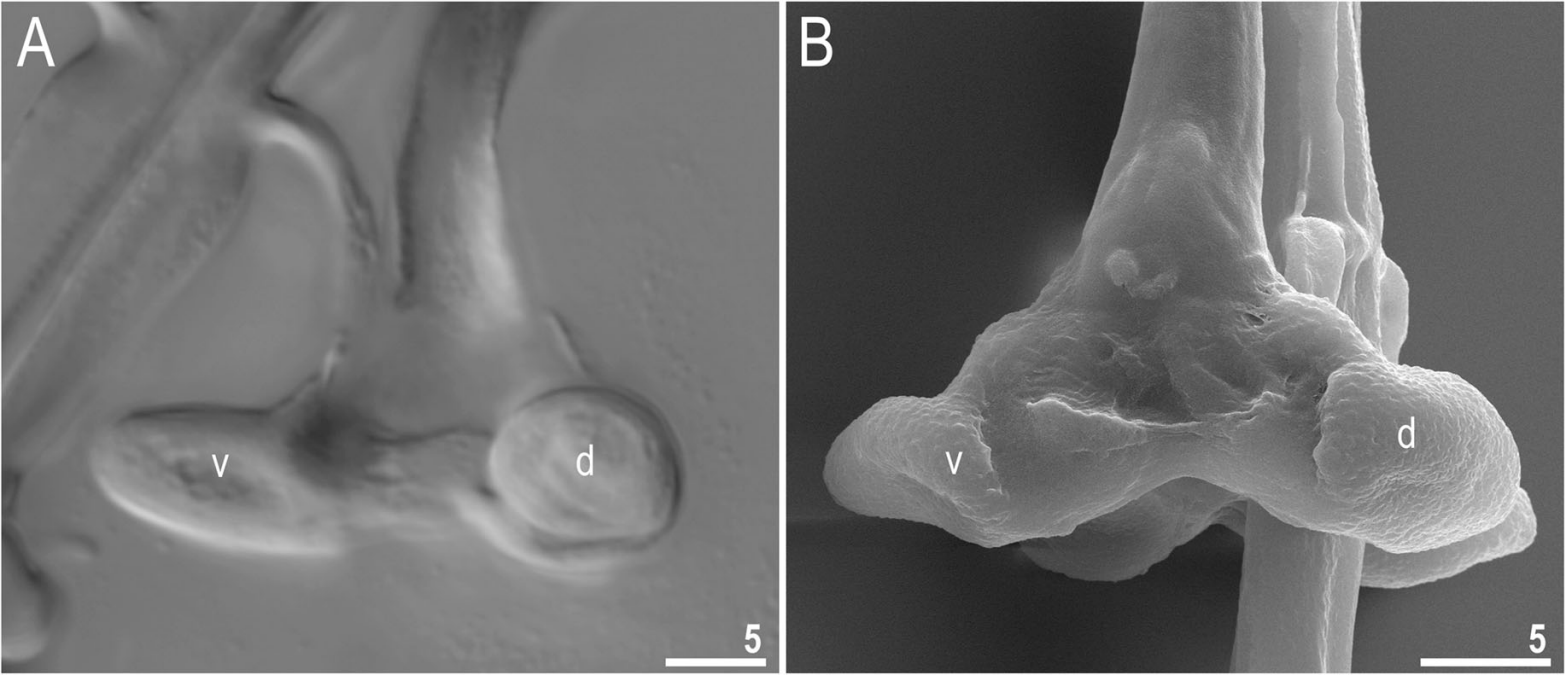
*Richtersius coronifer s.s.* (Richters, 1903) from the Billefjorden population, stylet furca. **a** Internal surface of furcae seen in NCM. **b** External surface of furcae seen under SEM. The ventral and dorsal condyles are indicated by “v” and “d”, respectively. Scale bars in μm

**Fig. 7:**
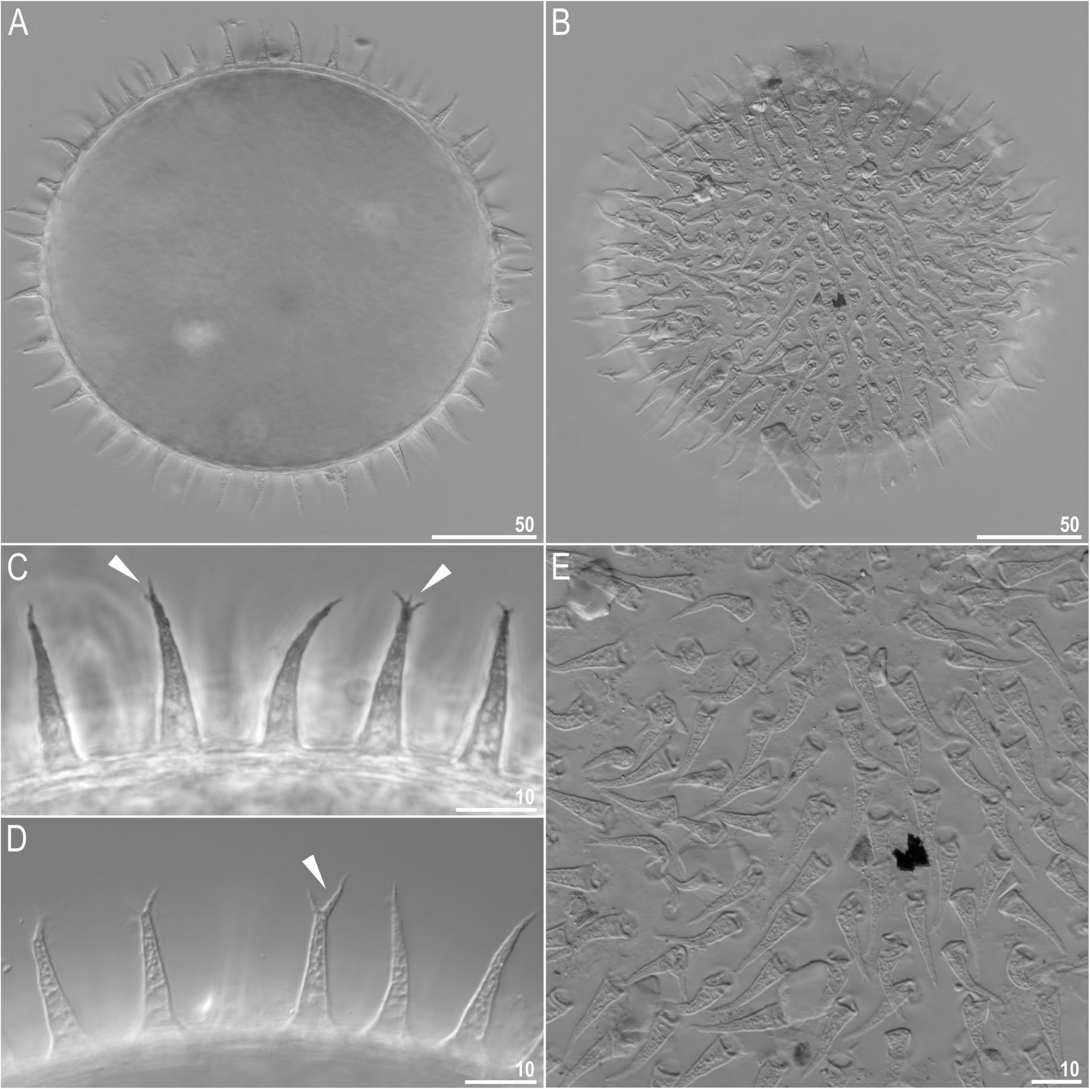
*Richtersius coronifer s.s.* (Richters, 1903) from the Billefjorden population, eggs seen in LCM. **a** Midsection under 400× magnification seen under NCM. **b**. Surface under 400× magnification seen under NCM. **c–d** Midsection under 1000× magnification seen in PCM and NCM, respectively. **e**. Surface under 1000× magnification seen in NCM. Arrowheads indicate divided process apices. Scale bars in μm

**Fig. 8:**
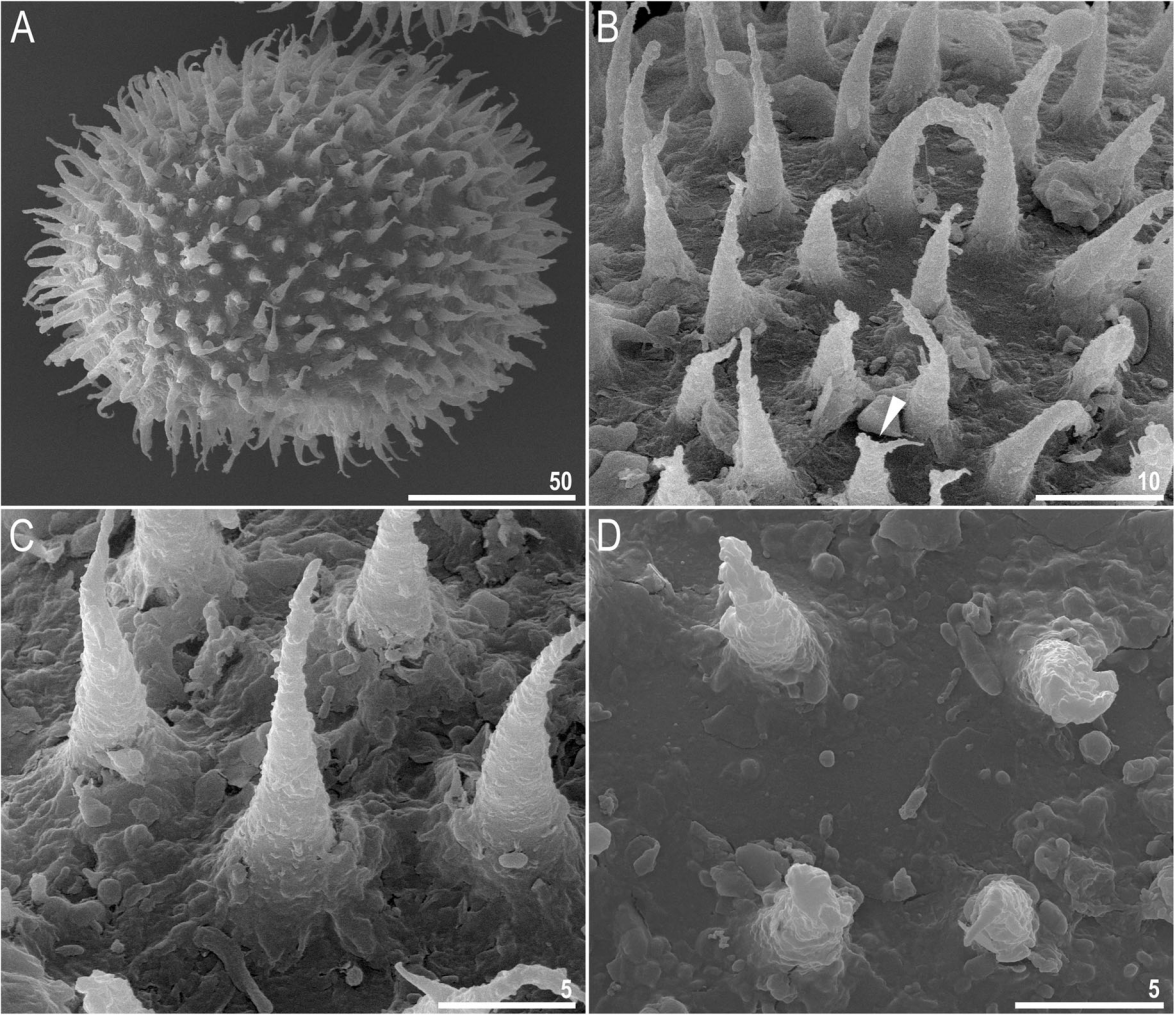
*Richtersius coronifer s.s.* (Richters, 1903) from Billefjorden population, egg chorion morphology seen under SEM. **a** Entire egg. **b**, **c** Details of processes. **d** Details of surface. Arrowhead indicate divided process apices. Scale bars in μm

*Macrobiotus coronifer* [13, 14]; *Adorybiotus coronifer* (Richters, 1903) [15]; *Richtersia coronifer* (Richters, 1903) [16]; *Richtersius coronifer* [17]

**Material examined:** 391 animals, and 248 eggs: specimens mounted on microscope slides in Hoyer’s medium (307 animals + 233 eggs), fixed on SEM stubs (20 + 15), processed for DNA sequencing (four animals) and aceto-orcein staining (60 animals).

### Redescription of *Richtersius coronifer* (Richters, 1903)

#### Animals (measurements, including the specimen proposed as the neotype, and statistics in Table 4)

Body intensively yellow in all instars, after fixation in Hoyer’s medium all specimens become transparent (Fig. 1a–b). Eyes present in live animals and in specimens mounted in Hoyer’s medium. Body and leg cuticle without any granulation in all instars (Fig. 1a–d). Round and oval pores (0.35–0.69 μm in diameter), clearly visible both under LCM and SEM, scattered randomly on the entire body cuticle only in hatchlings (Fig. 1b–d). In adults, cuticle poreless.

Claws slender, primary branches with distinct accessory points well visible under LCM (Fig. 2a–b). Secondary branches ca. twice as short as the primary branches. An evident stalk system connecting the claw to the lunule is visible under LCM and well visible under SEM. It consist of a thin laminar stalk connecting the claw to lunule and two posterior lateral expansions, whose distal tips under LCM seem to be connected to the stalk where it comes in contact with the lunule (Fig. 2a–b) whereas under SEM the stalk system is visible as a cuticular plate with a protuberant laminar stalk (Fig. 2c–f). Lunules very big, smoothly unified with cuticle of leg, with a crown of long, numerous and densely arranged spikes (2.5–3.2 μm long) (Fig. 2a–f). Lunules I–III trapezium-shaped, whereas lunules IV ovoid (Fig. 2a–f).

Mouth antero-ventral. Buccal apparatus massive. Sensory lobes merged into a single circular sensory field surrounding the mouth (Fig. 3a). Anteriorly, mouth begins with fused peribuccal lamellae forming circular velum/lamina which is posteriorly folded into a pre-mouth ventricle (Fig. 3a–b). Oral cavity armature is composed of three bands of teeth visible only under SEM (Fig. 3a–b). The first and the second band form continuous rings around the axis of the mouth, whereas the third band is divided into a dorsal and a ventral portion (Fig. 3a–b). The first band of teeth lays on the inner surface of velum and is composed of numerous small granular cones forming about 20 irregular rows with slightly bigger teeth laying closer to the edge of the velum (Fig. 3a–b). The second band of teeth consist of about 10 irregular rows of densely packed, elongated and sharp cones which lay on a cuticular fold protruding from the pre-mouth ventricle (Fig. 3a–b). The third band of teeth is situated between the second band of teeth and buccal tube opening and is discontinuous, divided into the dorsal and the ventral portion, both in the form of single, large teeth (Fig. 3a–b). The ventral tooth resembles an isosceles trapezium standing on its longer base, with a ragged upper edge. The dorsal tooth is semicircular in shape, with a crescent-shaped indentation in the middle, and can be only occasionally seen in LCM (Fig. 4c).

The oral cavity is followed by a system of massive apophyses forming a buccal crown (Figs. 4a–c, 5a–d). Anteriorly, the system consists of two triangular apophyses, one dorsal and one ventral (Fig. 5c–d). The dorsal, T-shaped apophyses, are composed of an anteriorly positioned large cuticular hook followed by a longitudinal crest (Figs. 4c, Fig. 5a–c). Ventrally, an analogous structure is formed by the ventral lamina, which begins anteriorly with bulbous apophysis similar to but smaller than the dorsal hook (Figs. 4a–b, Fig. 5b–d). Buccal tube wall exhibits differential thickness but the internal diameter of the buccal tube is almost constantly narrow (Fig. 4a). From mouth opening to stylet support insertion point, the thickness of the buccal tube wall grows slightly to quickly expand to the largest thickness after this point and then it shrinks posteriorly. Stylet supports lead to massive furcae, each composed of two rugged condyles, ventral and dorsal, forming together an arc. The dorsal condyle is strongly bent anteriorly, whereas the ventral condyle is bulbous (Figs. 5a, Fig. 6a). Both condyles are slightly folded into the direction of mouth opening (Fig. 6b). Pharynx spherical, with bilobed apophyses, three anterior cuticular spikes (typically only two are visible in any given pane) and two granular macroplacoids. The first and the second macroplacoid with a constriction positioned anteriorly and subterminally, respectively (Fig. 4d–e, 5e–f). The macroplacoid length sequence is 2 < 1.

#### Eggs (measurements and statistics in Table 5)

Big, oval, light yellow, laid freely. The surface between processes smooth but difficult to observe because of the amount of debris that is typically attached to the egg surface (Figs. 7a–b, e, Fig. 8a–d). Processes are in the shape of elongated, thin, conical spikes with ragged surface. Processes are internally reticulated (Fig. 6c–d). Ends of processes sometimes divided into two or three filaments (Figs. 6d, Fig. 7b). Terminal discs or spatulas absent.

#### Reproduction

Among 60 specimens stained with aceto-orcein, 18 males were found, thus the Billefjorden population is dioecious (gonochoristic-amphimictic).

#### Diet

Guts of individuals extracted from moss samples were always dark brown. This, together with the peculiar morphology of the oral cavity armature (numerous conical teeth placed in a shallow anterior portion of the cavity with beak-like teeth of the third band) and the anatomy of the buccal tube (thick walls and massive apophyses for muscle attachments), suggests that the species may feed on detritus scrubbed off from a surface.

##### Etymology

Richters [13] named the species “*coronifer*”, meaning “bearing a crown”, which refers to the wreath of spikes on each lunula that resembles a crown.

##### Locality

78°38′13″N, 16°46′07″E; 15 m asl: Norway, Svalbard, Spitsbergen, Brucebyen, Billefjorden (= Klaas-Billen Bay); tundra; moss on soil; coll. Collected 7 July 2017 by Michala Bryndová.

##### Slide depositories

specimen which will be proposed as neotype (Stec et al. in prep, Fig. 1a, slide NO.385.42 with 3 other specimen) and 306 specimens (slides: NO.385.*, where the asterisk can be substituted by any of the following numbers 01; 09–17; 28–30; 35–61; 89–95) and 233 eggs (slides: NO.385.*: 02; 06–08; 18–22; 49–55; 82–88) are deposited at the Institute of Zoology and Biomedical Research, Jagiellonian University, Gronostajowa 9, 30–387, Kraków, Poland and 30 specimens (slides: NO.385.*: 59–61) and 16 eggs (slides: NO.385.*: 54–55) are deposited in the Natural History Museum of Denmark, University of Copenhagen, Universitetsparken 15, DK-2100 Copenhagen Ø, Denmark.

### Genetic characterisation of the Billefjorden population

We obtained DNA sequences for all four of the above mentioned genetic markers. All of them were represented by single haplotypes: 18S rRNA, 1030 bp long; 28S rRNA, 784 bp long; ITS-2, 444 bp long; COI, 636 bp long. GenBank accession numbers for all these DNA sequences are provided in Table 5 together with accession numbers for other populations found in this study.

### Genotypic differential diagnosis

The ranges of uncorrected genetic p-distances between the Billefjorden population of *R. coronifer s.s.* and genotyped populations/species of the genus *Richtersius* are as follows (please see Additional file 3 for all values of the genetic distances):

- **18S rRNA**: 0.0–1.6% (0.8% on average), with the most similar being *Richtersius* sp. 2 (“*R.* cf. *coronifer*”) from Mongolia (KT778708–10), and the least similar being “*R. coronifer*” from Greenland (EU266931);
- **28S rRNA**: 3.5–4.2% (3.7% on average), with the most similar being *Richtersius* sp. 4 (IT.120, PL.246) from Italy and Poland (MH681758–9), and the least similar being *Richtersius* sp. 7 (GR.008) from Greece (MK211384);
- **ITS-2**: 20.4–21.9% (21.2% on average), with the most similar being *Richtersius* sp. 4 (PL.246) from Poland (MH681765), and the least similar being *Richtersius* sp. 6 (IT.317) from Italy (MK211382–3);
- **COI**: 0.2–21.9% (14.1% on average), with the most similar being *R. coronifer s.s.* from Greenland (KT778692–4), and the least similar being *Richtersius* sp. 7 (GR.008) from Greece (MK214323–4).

### Phylogeny and genetic species delimitation

**Fig. 9:**
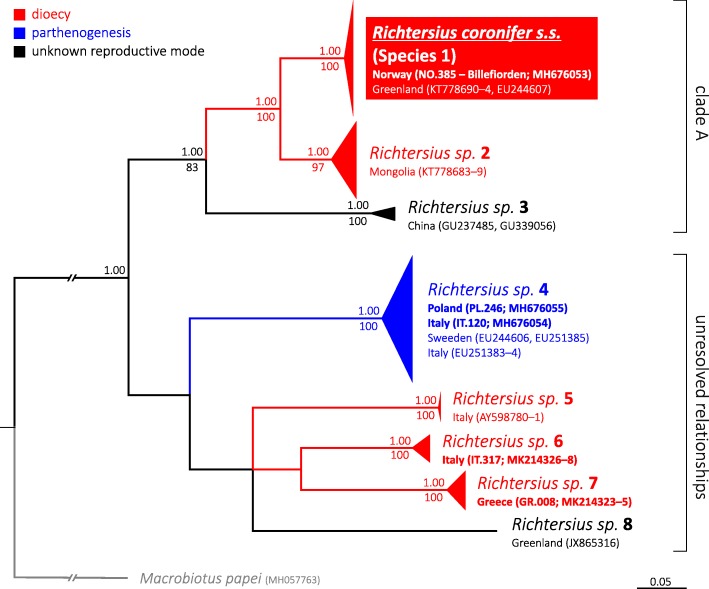
The *Richtersius* phylogeny constructed from COI sequences obtained in this study and from sequences available from GenBank. Numbers above the nodes indicate Bayesian posterior probability whereas the numbers under the nodes show bootstrap support values. The Bayesian Inference and Maximum Likelihood tree topologies were identical, thus only the Bayesian tree is shown. The Billefjorden population (which is proposed as new neotype population) is underlined whereas other newly found populations and new sequences are bolded. Please see “Comparative molecular analysis” and “Phylogenetic analysis” subsections in Material and Methods for details on sequences used in the analysis. The outgroup is marked with grey font and branches. The scale bar represents substitutions per position

The conducted phylogenetic analysis resulted in trees with a stable topology for eight species, with no topological differences between the ML and the BI analysis (Fig. 9). Using the criterion of reciprocal monophyly, eight clearly separated terminal clades with evidently longer interspecific than intraspecific branches were identified (Fig. 9). The node (supported only by BI analysis), that differentiates *M. papei* from all *Richtersius* populations, is polytomous with six branches: clade A (with three terminal nodes comprising *R. coronifer s.s*. from the Billefjorden population and Species 2–3), and Species 4–8 (Fig. 9). Generally, tree nodes were better supported in the BI than in the ML analysis. As there was a considerable polytomy on the presented COI tree, definite conclusions about the relationships between the putative species cannot be currently made.

The Maximum Likelihood and Bayesian solutions in the PTP analysis supported all eight terminal clades recognised in both phylogenetic analyses as separate species. The estimated number of species ranged from eight to 26, with the most conservative solution of eight species being the most supported. Although the PTP analysis of ML tree obtained in this study suggested that the two *Richtersius* sequences from China are separate species, the support for this recognition was small (support = 0.59), thus the most conservative solution of eight species was most probable. The AGBD analysis also always returned eight separated groups, as the most supported result of the delimitation. Moreover, the distribution of uncorrected genetic distances showed a wide barcoding gap and a considerable genetic divergence (9.8–24.2%; 20.1% on average) between each of the eight putative species, with divergences within species being very low (0.0–2.0%; 1.0% on average; please see Additional file 3 for details). The results based on the mitochondrial marker COI were additionally supported by the considerable genetic divergences in the nuclear marker ITS-2 also between the four species sequenced in this study. Genetic distance in ITS-2 between the four species ranged from 7.1 to 21.9% (14.9% on average), whereas within species they varied from 0.0 to 0.5% (0.3% on average; please see Additional file 3 for details).

### Geographic distribution of the genus *Richtersius*

The eight putative species analysed in the present study, i.e. represented by GenBank sequences and sequences obtained from new populations, comprised records from the Palaearctic and the Sino-Japanese realm, i.e., exclusively from Eurasia. There was no evident geographic pattern of clustering that could be seen on the phylogenetic tree (Fig. 9). The tree comprises two species (Species 1 and 8) from the Arctic, two species (Species 2 and 3) from Asia and four remaining species (Species 4–7) from Europe (Fig. 9). Moreover, six of the eight putative species were found only once and the two remaining species (*R. coronifer s.s.* and Species 4) were collected from 2 to 4 localities. Specifically, genetically verified records of *R. coronifer s.s.* (Species 1) are limited to the Arctic (Svalbard and Greenland) whereas Species 4 was collected from four European localities representing three countries (Italy, Poland and Sweden), which makes it the most widespread currently known *Richtersius* species (Fig. 9).

### Tests of morphological character for phenotypic species delimitation in *Richtersius*

**Fig. 10:**
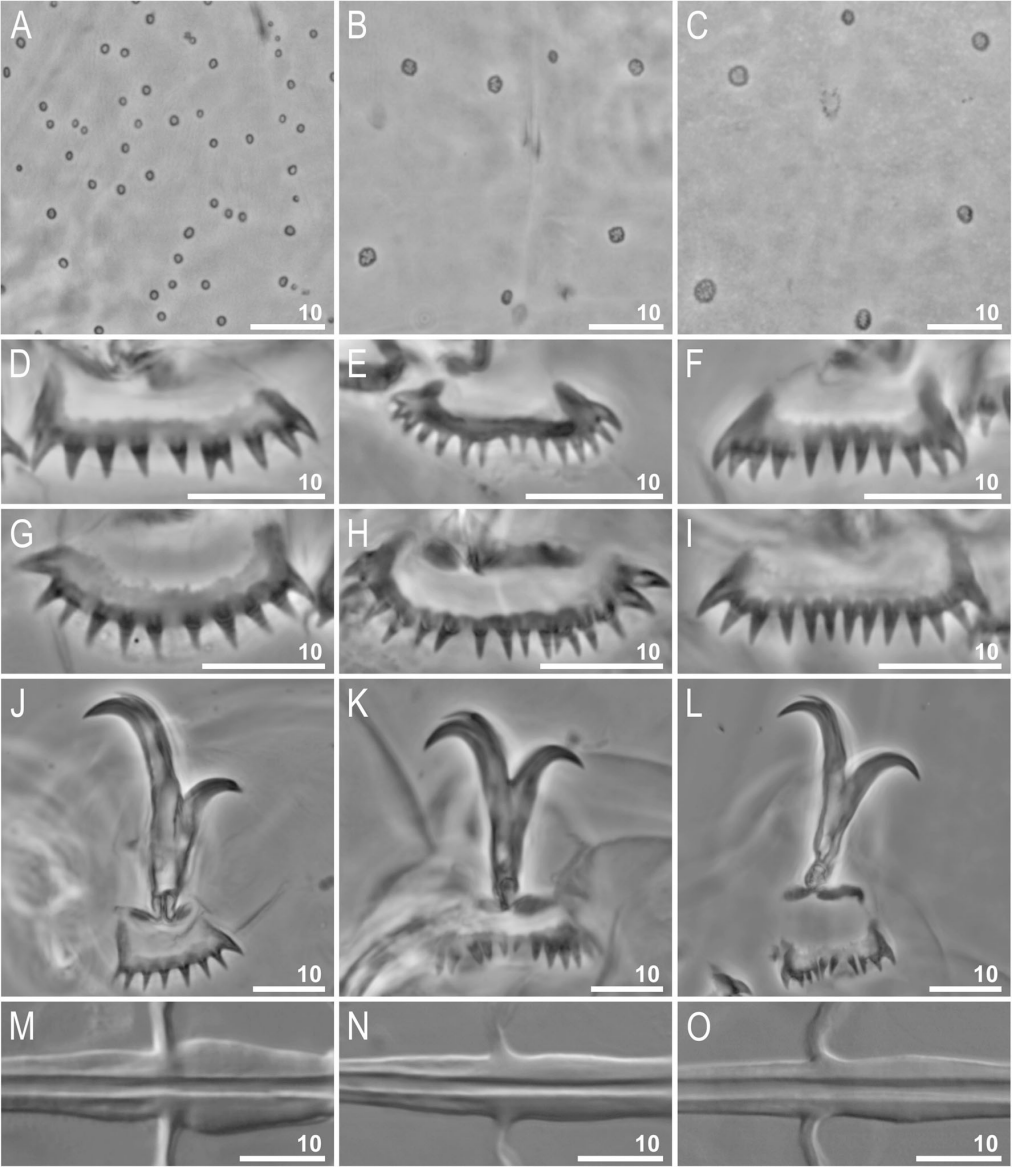
A comparison of three exemplars from among the eight *Richtersius* species genetically delimited in the present study. Shown are PCM photomicrographs of morphological traits which could be useful in their differentiation. **a**–**c** Cuticular pore morphology. **d**–**f** External lunule III morphology. **g**–**i** Posterior lunule morphology. **j**–**l** Claws III (internal, external and internal, respectively). **m**–**o** Morphology of the buccal tube walls around the level of the stylet insertion point. The first column presents *Richtersius coronifer s.s.* (NO.385) from the Billefjorden population, the second and third columns show *Richtersius* sp. from Italy (IT.120) and Greece (GR.008), respectively. Scale bars in μm

The ANOVAs for pore density, pore size, and number of teeth on the external and internal lunules III and on the anterior and posterior lunules IV showed statistically significant differences in all traits between the three analysed species (**PD**: F_2, 27_ = 628.2; *P* < 0.001; **PS**: F_2, 270_ = 376.34; *P* < 0.001; **ExtT**: F_2, 27_ = 25.9; *P* < 0.001; **IntT**: F_2, 27_ = 15.8; *P* < 0.001; **AntT**: F_2, 27_ = 32.7; *P* < 0.001; **PosT**: F_2, 27_ = 44.5; *P* < 0.001; **SSIP**: F_2, 27_ = 111.6; *P* < 0.001; **CCT**: F_2, 27_ = 19.2; *P* < 0.001; see Table 6 for ranges, means and standard deviations). Specific comparisons with Tukey post-hoc testing for pore density (PD) and teeth on internal lunules III (IntT) showed significant differences between the *R. coronifer s.s.* from the Billefjorden population (NO.385) and the two other analysed species, i.e. *Richtersius* sp. 4 from Italy (IT.120) and *Richtersius* sp. 7 from Greece (GR.008), whereas there were no differences between the GR.008 and the IT.120 population. For pore size, *pt* of the stylet support insertion point, and claw common tract index, Tukey post-hoc showed significant differences between all populations. The *R. coronifer s.s.* from the Billefjorden population (NO.385) was characterised by significantly smaller and more densely distributed pores as well as by a smaller number of teeth on the internal lunules III compared to the south European species. Moreover, the cuticular pores of the *R. coronifer s.s.* from the Billefjorden population (NO.385) had smooth rims whereas pores of the Italian and the Greek species (IT.120 and GR.008, respectively) had jagged margins (Fig. 10a–c). The *R. coronifer s.s.* from the Billefjorden population (NO.385) had the most posteriorly positioned stylet supports, followed by Species 4 (IT.120) and then Species 7 (GR.008), but it also had evidently thicker buccal tube wall posterior to the stylet support insertion point compared to Species 4 and 7 (Fig. 10m–o). *R. coronifer s.s.* (NO.385) had also the longest primary branches compared to claw common track followed by Species 7 and 4 (GR.008 and IT.120, respectively) with the last one having the shortest braches (Fig. 10j–l).

**Table 6:** Means with standard deviations and ranges of putative new morphometric characters for phenotypic species delimitation in *Richtersius* tested in this study

Species (population)	statistic	PD	PS	ExtT	IntT	AntT	PosT	SSIP	CCT
*R. coronifer s.s.* (NO.385)	mean ± SD	74 ± 7	1.2 ± 0.2	11 ± 1	10 ± 1	10 ± 2	11 ± 1	73.6 ± 1.2%	49.6 ± 4.4%
	range	60–88	0.7–1.6	9–11	8–12	9–13	10–11	72.2–75.6%	42.5–55.7%
*Richtersius* sp. 4 (IT.120)	mean ± SD	10 ± 2	2.1 ± 0.3	14 ± 2	13 ± 1	18 ± 3	16 ± 1	68.7 ± 1.3%	61.8 ± 3.4%
	range	6–13	1.3–3.0	12–17	11–15	14–22	14–19	66.8–70.8%	57.7–68.4%
*Richtersius* sp. 7 (GR.008)	mean ± SD	6 ± 2	2.9 ± 0.6	13 ± 1	12 ± 1	12 ± 2	13 ± 1	64.7 ± 1.3%	56.3 ± 4.6%
	range	4–10	1.6–4.2	11–14	10–15	9–15	11–15	63.3–66.4%	46.8–65.8%

*PD* the number of pores within rectangle of 2500 μm^2^ of dorsal cuticle between legs III and IV (hatchlings, i.e. first instars only); *PS* – pore size, measured as the longest diameter (μm); *ExtT, IntT* – the number of teeth on external and internal lunules III (adults, i.e. instars 2+ only), respectively; *AntT, PosT* – the number of teeth on anterior and posterior lunules IV, respectively (adults only); *SSIP* – *pt* of the stylet support insertion point (expressed as percentage); *CCT* – claw common tract index for external claws III (expressed as percentage). Ten specimens have been measured for each trait. For PS, 10 pores each from each of the 10 specimens have been measured

Significant differences between all examined populations were also recovered in specific comparisons of numbers of teeth on internal lunules III and posterior lunules IV (IntT, PosT) with the *R. coronifer s.s.* from the Billefjorden population (NO.385) being characterised by the smallest and the Italian *Richtersius* sp. 4 (IT.120) by the largest number of teeth. Finally, for the number of teeth on the anterior lunules IV, the Tukey post-hoc testing recovered significant differences between *R. coronifer s.s.* (NO.385) and *Richtersius* sp. 4 (IT.120) and also between *Richtersius* sp. 4 (IT.120) and *Richtersius* sp. 7 (GR.008), whereas there were no differences between the *R. coronifer s.s.* from the Billefjorden population and the Greek *Richtersius* sp. 7 (NO.385 and GR.008, respectively). Please see the supplementary material (Additional file 1) for *p*-values for each specific comparison with Tukey post-hoc test.

### Reproductive modes

Three of the four genetically delimited *Richtersius* species represented by the five newly found populations analysed in this study were dioecious (gonochoristic-amphimictic). Aceto-orcein staining revealed males with testis filled with sperm in *R. coronifer s.s.* (the Billefjorden population NO.385). Moreover, although the Greek and the Sardinian population (IT.317 and GR.008; *Richtersius* sp. 6 and 7, respectively) were not subjected to aceto-orcein staining, three and fifteen males (testis with sperm) were observed among animals freshly mounted in Hoyer’s medium in both these populations respectively. On the other hand, no males were found among freshly mounted individuals in the two remaining populations, IT.120 and PL.246, representing *Richtersius* sp. 4, what indicates that most likely the species is parthenogenetic. Therefore, our analysis shows that the genus *Richtersius* comprises both dioecious and clonal species.

### Transcriptome characteristics

**Fig. 11:**
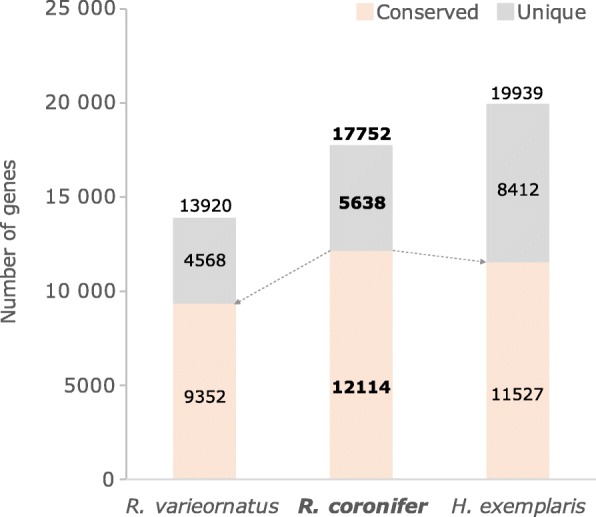
A comparison of gene repertoire of *R. coronifer s.s.* with previously published genomes of *R. varieornatus* and *H. exemplaris*. BLASTX queries were run with the *R. coronifer s.s.* transcriptome as input against the proteome set in the two genomes, with e-value threshold of 1e-5. The number of conserved genes (9352–12,119) as well as their percentage within the total proteome (57–68%) were highly consistent throughout the analysed transcriptomes and genomes. These set of genes include those related to anhydrobiosis, including many tardigrade-specific heat soluble proteins

Transcriptome assembly resulted in 21,091 transcripts, where 17,752 were unique transcripts without isoforms. Completeness of the assembly assessed by BUSCO score was 86.8% (Complete + Partial), indicating sufficient coverage of genes for a single-condition and single-specimen sample. Detailed statistics of assembly is shown in Table 7. Of the 17,752 unique transcripts, 12,114 yielded a hit in *R. varieornatus* or *H. exemplaris* proteins by BLASTX searches with 1e-5 threshold. These conserved genes correspond to 9352 and 11,527 genes in *R. varieornatus* and *H. exemplaris*, respectively Fig. 11). The number and percentage of conserved genes are highly consistent throughout the three Parachela datasets. When such conservation analysis was extended to Arthropoda (*D. melanogaster*) and Nematoda (*C. elegans*), we see that of the around 10,000 genes conserved among Parachela, around 6000 are conserved among ecdysozoans, and around 3000 are tardigrade-specific. These conserved genes included tardigrade-specific anhydrobiosis-related genes such as CAHS, SAHS, MAHS, but – similarly to *H. exemplaris* – Dsup [90–92] was not found in *R. coronifer s.s.* (see the annotated BLAST result against *R. varieornatus* reference proteome uploaded at FigShare 10.6084/m9.figshare.8797184 for details)*.*

**Table 7:** Summary statistics of *Richtersius coronifer s.s.* transcriptome assembly

Transcriptome	
Total assembled transcripts	21,091
Total assembled length	13,304,280 bp
Average transcript length	630 bp
Longest transcript length	7623 bp
Shortest transcript length	201 bp
N50 (number of transcripts in N50)	931 bp (4741)
N90 (number of transcripts in N90)	267 bp (15,226)
BUSCO (Eukaryota database)	
Complete BUSCOa	60.40%
Complete + Partial BUSCOs	86.80%
Missing core genes	13.20%
Average number of orthologs per core genes	1.26
Genes	
Total number of transcripts	21,091
Unique transcripts (excluding isoforms)	17,752
Unique transcripts with BLAST matches to tardigrade genomes (E-value < 1.0e-5)	12,114

## Discussion

The present study explicitly reveals hidden species diversity within the genus *Richtersius*. At first, by proposing the neotype reinstatement and integrative redescription of *Richtersius coronifer* (Richters, 1903) [13], the taxonomic obstacle caused by the out-dated and incomplete species description of the nominal species and the uncertain neotype designation by Maucci & Ramazzotti [15] may now be removed. With the detailed morphological and molecular data presented for the redescribed nominal taxon from Billefjorden, i.e. one of the original localities studied by Richters [13], the species diversity within the genus *Richtersius* can now be explored much further and deeper. Secondly, using the molecular species delimitation methods we have confirmed the previous hypotheses on the presence of more than one species under the name “*Richtersius coronifer*” [18–20], showing that at least seven new putative species are present within the genus. Finally, the redescription of *R. coronifer s.s.* was aided, for the first time in tardigrade taxonomy, by transcriptome data, the use of which will facilitate future studies on the genus.

*Richtersius coronifer* was reported by earlier researchers from the Arctic, Europe, North and South America, Africa and Asia, i.e. from the following six zoogeographic zones (zonation according to Holt et al. [93]): Palearctic, Nearctic, Saharo-Arabian, Afrotropical, Neotropical and Sino-Japanese realm [94–97]. The majority of such records consistently came from cold regions, i.e., from the Arctic and Sub-arctic regions or mountainous areas on other continents. However, our analysis of 13 populations from Eurasia and Arctic indicated as many as eight potential species in the genus (0.6 species/population on average) of which all could be identified as *R. coronifer* since the original description of *Richtersius coronifer* is very basic. Thus, in the light of these findings, all records of “*R. coronifer*”, except for the original account by Richters [13], the records from Greenland by Guidetti et al. [18] and Schill & Jönsson (unpubl.), and the Billefjorden population from Svalbard described in this study (population NO.385), should be designated as a “*R.* aff. *coronifer*” unless positively verified with the data presented here. In fact, other alleged records of the species from the Arctic should also be designated as a “*R.* aff. *coronifer*”, because our study showed that a Greenland population sequenced and identified by Sands et al. [66] as “*R. coronifer*” represents another putative new *Richtersius* species (*Richtersius* sp. 8; see Fig. 9). Interestingly, we have found also that in Italy, three distinct putative species are present. This additionally supports our solution with proposed neotype reinstatement, as it is still probable that the neotype established by Maucci & Ramazzotti [15] with a population from a more southern locality in continental Norway did not represent *R. coronifer s.s*., but a different species. In other words, all earlier records of “*R. coronifer*” from Europe, North and South America, Africa and Asia are evidence for the geographic range of the genus, but not of the nominal species, which has a confirmed geographic distribution spanning from Svalbard to Greenland. This does not, of course, mean that *R. coronifer s.s*. has a geographic range limited to the Arctic. It only means that the currently available genetic data for the genus do not allow for the inclusions of any other *Richtersius* records to be identified as *R. coronifer s.s*.

Our genetic species delimitation conducted on COI sequences of only thirteen populations from Europe and Asia revealed a considerable diversity of eight putative species. Single locus delimitation should be treated with caution [98], but on the other hand it is a very useful tool that allows to formulate Primary Species Hypothesis [85] that can be a sound starting point for deeper studies [7]. Recently, a considerable discrepancy in the genetic distances between COI and ITS-2 of different species in the genus *Milnesium* Doyère, 1840 [86] and *Paramacrobiotus* Guidetti, Schill, Bertolani, Dandekar, Wolf, 2009 [99] have been discovered, making single-locus genetic delimitation more challenging [100, 101]. However, in our study on the genus *Richtersius*, genetic distances between the four species, for which both COI and ITS-2 sequences were available, gave congruent delimitative results in both markers, indicating that both of them could be useful in further delimitation studies, at least in this tardigrade group.

Although we confirmed the presence of hidden diversity within the genus *Richtersius*, the question of whether the putative species genetically delimited in the present study are cryptic or pseudocryptic, as previously suggested [18–20], remains open. Similarly to Guidetti et al. [18], we found that some species may differ by the morphology of buccal tube walls, claws and cuticular pores. However, currently only for the latter trait are the distinct states known (smooth vs. jagged margins). Beside these, we also did not note other obvious morphological differences between the three morphologically analysed species. Nevertheless, we suspect that SEM imaging may provide more detailed information about morphology of pores and possibly also about details of egg chorion ornamentation. Importantly, our preliminary morphometric examination of the five new morphological traits and statistical comparisons showed a number of statistically significant differences between the three analysed species. Although the sample size (*N* = 10 per species) in these comparisons was limited due to the small number of individuals found in populations other than the Billefjorden population, it seems that these traits with increased sample size may be useful and valuable in future delimitations of *Richtersius* species. However, in the future taxonomic studies on the genus *Richtersius*, the resolution of analysed data should be extended, for example, by rigorous statistical comparisons of detailed morphometric data [100], geometric morphometrics [102], ITS-2 secondary structure comparisons [103], karyotyping, reproductive mode analysis [11], and experimental inter-population crosses [101].

Interestingly, of six putative species for which the reproductive mode is known (i.e. all species except *Richtersius* sp. 3 and 8), one is parthenogenetic (*Richtersius* sp. 4; 18, 19, 20, present study) and the remaining five are dioecious: *R. coronifer s.s.* ([18], present study), *Richtersius* sp. 2 [18], *Richtersius* sp. 5 [19, 62], *Richtersius* sp. 6 (present study), and *Richtersius* sp. 7 (present study). Thus, according to currently available data, the genus is predominantly dioecious, and parthenogenesis, being present in only one phyletic lineage, possibly evolved once within the genus. Among all genetically delimited species in our study, the parthenogenetic *Richtersius* sp. 4 was the most often sampled as it was found in four European localities, with the maximum distance between populations of ca. 2430 km. This results seem to be in line with recent findings on the *Paramacrobiotus richtersi* complex by Guidetti et al. [11] who found that a parthenogenetic species had a seemingly wider distribution compared to other dioecious species analysed in their study. However, it should be also noted that *R. coronifer s.s.*, a dioecious species, was found in two Arctic localities which are separated by a comparable distance of ca. 2220 km. Therefore, with such a limited number of analysed populations both in Guidetti et al. [11] and in the present study as well as with the very restricted phylogenetic sampling, it is too early to draw general conclusions on the effects of reproductive mode on tardigrade species dispersal and distribution.

Transcriptome assembly of *R. coronifer s.s*. along with the redescription in this work provides a molecular foundation for further study of this species. The assembly is currently based on a single-specimen method to minimise contamination, so the expressed transcripts is not necessarily comprehensive, and BUSCO completeness assessment actually shows that many of the assembly is still partial. On the other hand, complete + partial BUSCO coverage is 86.8%, which is sufficiently high to look into the gene repertoire in this species. While this manuscript was under review, another transcript assembly of a *Richtersius* species collected in Europe, identified as *Richtersius* cf. *coronifer*, was published by Kamilari et al. [40]. Upon inspection of the COI sequence included in this assembly, this species was identified as *Richtersius* sp. 4 in our phylogenetic analysis (Fig. 9). However, the assembly included also a small fraction of further five alien COI sequences (a total of 319 alien vs 470,000 *Richtersius* sp. 4 reads), namely: CL1502.Contig2_Richtersius (matches EU244606.1 *Richtersius* sp. 4), CL3227.Contig2_Richtersius (matches MK430674.1 *Penes monodon* shrimp, e-value = 0.0, 99.9% identity), CL3931.Contig3_Richtersius (matches AY508520.1 *Bicycles funebris* butterfly, e-value = 0.0, 97.1% identity), Unigene12939_Richtersius (best hit to KJ669420.1 *Gadopsis bispinosus* fish, e-value = 9e-32, 88.0% identity), Unigene16155_Richtersius (matches to MK217264.1 *Homo sapiens*, e-value = 0.0, 100.0% identity), 6. Unigene17913_Richtersius (matches to KX071951.1 *Biston betularia* moth, e-value = 1e-144, 100.0% identity). This indicates cross contaminations during Illumina multiplexing. Furthermore, isoform clustering of Kamilari et al. [40] assembly does not seem sufficient. For example, 22,453 “Unigenes” of the assembly match 11,244 *R. varieornatus* genes with BLASTX with e-value threshold of 1e-5, and likewise there are 22,922 matches to 14,253 in *H. exemplaris*. Similarly, 20,744 of their assembly matches 14,474 genes of our assembly, suggesting the presence of × 1.4~ × 2.0 unclustered isoforms. Moreover, the filtering process in Kamilari et al. [40] seemed to lose some of the genes (e.g., of the 116 genes that match *R. varieornatus* reference proteome in our assembly, 51 can be rescued by re-assembling Kamilari et al. [40] transcriptome ab initio from raw reads. Therefore, due to different assembly parameters, tools and thresholds of contigs redundancy removal etc., a direct comparison between the two assemblies is beyond the focus of this work.

**Fig. 12:**
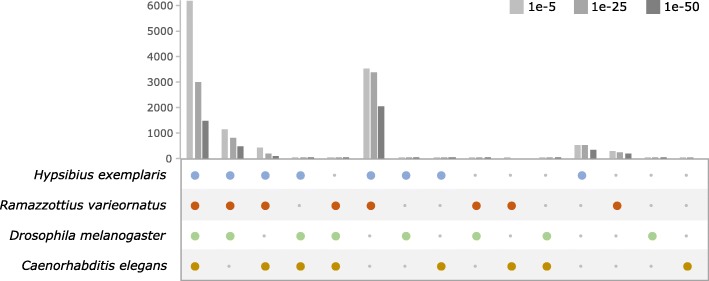
A comparison of gene repertoire among ecdysozoans, with *D. melanogaster* representing Arthropoda, and *C. elegans* representing Nematoda. Number of shared genes are shown as bar graphs at three different BLASTX thresholds (1e-5, 1e-25, 1e-50), and large coloured circles below the bars indicate the group of organisms where the genes are conserved. For example, the left most column indicates a conservation pattern among all four species tested, and the second column from the left indicates conservation in *H. exemplaris, R. varieornatus,* and in *D. melanogaster*, but not in *C. elegans*. Highest conservation at 1e-25 and 1e-50 are among tardigrades, due to their closer phylogenetic affinities

Comparisons of *R. coronifer s.s*. and *R. varieornatus*, and *H. exemplaris* gene sets, as shown in Fig. 12, illustrate high conservation of core gene sets within the class Eutardigrada. Of the 12,114 genes conserved between *R. coronifer s.s.* and either *R. varieornatus*, and *H. exemplaris*, 11,998 matched to 19,238 “Unigenes” of Kamilari et al. [40] assembly of *Richtersius* sp. 4 that was published during the review process of this paper, and 40 out of the 116 unmatched genes could be rescued by *ab initio* assembly of Kamilari et al. [40] raw reads, therefore the gene set within the genus *Richtersius* is highly conserved (99.4%). Conservation of tardigrade-specific anhydrobiosis-related proteins including CAHS, SAHS, MAHS, and LEAm identified in the two previously sequenced genomes of Hypsibioidea and in *R. coronifer s.s*. (see the annotated BLAST result at FigShare 10.6084/m9.figshare.8797184 for details) strongly suggests the acquirement of these genes at or before the common ancestor of Parachela. Again, the availability of transcriptome data in Macrobiotoidea opens new possibilities in comparative genomics of tardigrades.

## Conclusions

The integrative redescription of *R. coronifer s.s*. is likely to significantly affect the taxonomy of the genus and will open the window for species diversity exploration, as was previously shown for other nominal species redescriptions in tardigrades (e.g. [7–11, 61, 101]). Given that our genetic delimitation analysis of only 13 *Richtersius* populations revealed as many as eight potentially new species, we should expect species diversity in the genus to be largely underestimated, as it was also showed for other tardigrade groups (e.g. [7, 11, 100, 101, 104]). Finally, we strongly recommend that new *Richtersius* species should be described by means of integrative taxonomy in order to avoid future misidentifications and avoid the creation of new taxonomic obstacles. For example, if a new *Richtersius* species with a unique and distinct morphological trait that differentiates it from *R. coronifer s.s*. is found and described classically, it may turn out in the more distant future that more species exhibit the trait, but they are all morphologically very similar to each other. In such a case, with no DNA sequences, the descriptions of the other species may not be possible until the original species is integratively redescribed. In addition to light microscopy observations, SEM imaging and sequences of highly variable DNA markers (such as COI or ITS-2) should be incorporated in every new *Richtersius* description. However, if cryptic or pseudocryptic species are documented, other approaches, such as those mentioned in the previous paragraph, which allow for higher resolution analysis, should be considered. We also suggest that incorporating genomic data to descriptions and redescriptions of at least nominal taxa should be considered as good practice in taxonomic work. Last but not least, the fact that at the moment the genus *Richtersius* comprises only a single formally described species, for which we have proposed the redescription in this work, provides a unique opportunity for it to be the first tardigrade genus in which all species are described integratively.

## Supplementary information

**Additional file 1.** Raw measurements of additional morphological traits and computed statistics.

**Additional file 2. **Raw morphometric measurements underlying the proposed redescription of *R. coronifer s.s.*

**Additional file 3. **Uncorrected pairwise distances between *Richtersius* spp.

## Data Availability

All data generated or analysed during this study are included in this published article [and its supplementary information files]. Genetic, genomic and transcriptomic data are deposited in GenBank, NCBI SRA and FigShare, respectively.
